# High Corticosterone, Not High Energy Cost, Correlates with Reproductive Success in the Burrow-Nesting Ancient Murrelet

**DOI:** 10.1371/journal.pone.0084280

**Published:** 2013-12-31

**Authors:** Akiko Shoji, Kyle H. Elliott, Kathleen M. O’Reilly, Anthony J. Gaston

**Affiliations:** 1 Environment Canada, National Wildlife Research Centre, Ottawa, Ontario, Canada; 2 University of Manitoba, Department of Zoology, Winnipeg, Manitoba, Canada; 3 University of Portland, Department of Biology, Portland, Oregon, United States of America; 4 University of Ottawa, Department of Biology, Ottawa, Ontario, Canada; Institut Pluridisciplinaire Hubert Curien, France

## Abstract

Theory and observations suggest that offspring abandonment in animals may occur when the costs to future reproductive output of current reproductive effort outweigh the fitness benefits of rearing the current brood. While hormonal cues (i.e. corticosterone) or energy reserves are believed to be involved, few studies have directly focused on the proximate cues influencing behaviours directly related to reproductive success. To address this information gap, we determined the incubation metabolic rates and corticosterone (CORT) levels of naturally fasting and freely incubating ancient murrelets (*Synthliboramphus antiquus*). Respiratory quotient (RQ) increased with date, suggesting that incubating ancient murrelets shifted from strictly lipid-based metabolism towards more protein-based metabolism as incubation progressed. Birds that hatched only one nestling had higher levels of circulating CORT than those which hatched two, suggesting that birds which laid only a single egg found incubation more stressful than those which laid two. However, CORT levels and incubation shift lengths were not correlated, suggesting that birds that undertook prolonged incubation shifts did so only when their energy stores were not jeopardized.

## Introduction

Iteroparous animals face a trade-off between conserving their own energy and delivering energy to their young [1]. In particular, animals may abandon a current reproductive attempt when the associated cost is too high [1]. Many studies have focused on the ultimate mechanisms that mediate these evolutionary tradeoffs [2], [3], and some studies have examined the proximate cues underlying behaviours that determine reproductive success, such as abandonment (e.g., in long-lived seabirds: [4]).

Under unfavorable weather conditions, partners of incubating seabirds may extend their foraging trips to maintain their own body reserves while the incubating bird is fasting on the eggs [4], [5]. The incubating bird adjusts to prolonged fasting by mobilizing fat stores and sparing body proteins [6]. However, once a threshold is crossed, it is thought that protein metabolism also begins to contribute to energy production so that neither body proteins nor lipids are fully depleted; once mass reaches a lower threshold, the bird abandons [7]. A high metabolic rate–associated with large muscles and digestive tract–may increase a bird’s ability to capture food at-sea or adjust to changes in food distribution, but lead to increased reserve depletion at the colony [8], [9], [10], [11]. Thus, incubation metabolic rate (IMR) may play a strong role in regulating reproductive success, as birds with a higher metabolic rate (due to differences in thermoregulation, thyroid hormone levels or body composition) may abandon sooner than those with a lower rate.

A recent review suggested that resting metabolic rate is often correlated with individual behavior, especially aggressiveness, across a wide range of taxa [12]. Few studies, mainly of penguins and petrels, have examined the effect of metabolism on breeding behavior or success. Male burrow-nesting Leach’s storm-petrels (*Oceanodroma leucorhoa*), with relatively low basal metabolic rates, hatched their eggs earlier in the season and had higher offspring wing growth rates than males with relatively high basal metabolic rates; there was no effect on lifetime hatching success or female reproductive parameters [13]. In contrast, daily energy expenditure was not correlated with reproductive success (offspring feeding rates) in Brünnich’s guillemots (*Uria lomvia*), although those authors did not examine resting metabolic rate [14]. Given the possibility of linkages between metabolism, at-sea foraging success and incubation shift length, we hypothesized that metabolic rate might be a good predictor of reproductive success in seabirds.

Indirect calorimetry is a common method to estimate metabolic energy expenditure in animals, based on oxygen consumption rate (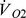) or carbon-dioxide production rate (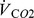). The respiratory quotient (RQ) of CO_2_ produced to O_2_ consumed is an index that characterizes energy sources of activity in a given period; low RQ (i.e. ∼0.7) indicates reliance on lipid substrates, whereas high RQ (i.e. ∼1.0) characterizes protein use. RQ may increase during long fasting periods, because the birds shift into phase III fasting, when protein catabolism increases [15], [16], [17]. However, available information for seabirds is limited to penguins and albatrosses, which have very long incubation shifts; species with shorter incubation shifts, like auks, have seldom been examined.

Baseline levels of corticosterone (CORT) were negatively correlated with reproductive success and body condition in black-legged kittiwakes (*Rissa tridactyla*; [18], [19]), as well as with food availability in common guillemots (*Uria aalge*; [20]). CORT levels correlate at an individual level with rates of abandonment in passerines ([21], [22]; see also [23]). We therefore predicted that CORT levels should increase with incubation shift length (low food supply requiring longer periods at sea) and should decrease with reproductive success (higher nest desertion rates being associated with lower food supply) [24], [25], [26]. Many of the processes involved in IMR, such as thermogenesis and basal metabolic rate, are controlled to a large degree by the thyroid hormones. Because CORT is involved in energy mobilisation, and can be a proximate cue or “refeeding signal” that triggers nest desertion when animals have depleted energy stores (e.g. [27], [28]), we also predicted that IMR may be positively correlated with reproductive success due to decreased energy metabolism and associated thermogenesis in birds prior to nest desertion. Specifically, CORT can exert a negative feedback on T3 to regulate energy homeostasis (e.g. [30]).

We examined the relationships between IMR, metabolic substrate, CORT, fasting duration and reproductive success. Using wild birds nesting in artificial nest boxes, we were able to measure the rates of oxygen consumption and carbon dioxide production of freely incubating small auks (ancient murrelets, *Synthliboramphus antiquus*) in the field without causing them any disturbance. We further tested whether longer fasting endurance was correlated with an increased respiratory quotient (RQ) through greater protein catabolism [29]. We believe that this is the first study to examine metabolic rate, incubation shift length and corticosterone levels in free-living birds.

## Materials and Methods

### Ethics Statement

Appropriate animal care permits were obtained (Environment Canada National Wildlife Research Centre Animal Care Permits: 0800AG02 [2008], 10AG02 [2010]).

### Study Site and Animals

Ancient murrelets are small (∼220 g) auks that share incubation equally between pair members [30]. The normal clutch size is two eggs, but a minority of birds lay only one egg [31]. Because incubation shifts are longer (2–3 days) than those of other auks, and because murrelets, unlike most seabirds, do not rear young at their nest [31], incubation is probably the most demanding phase of breeding in this species [6], [8], [32]. We measured plasma CORT levels of 38 ancient murrelets during April-June 2008 at Reef Island, Haida Gwaii, BC, Canada (52.52° N, 131.31°W). In April-May 2010 we returned to Reef Island to measure the incubation metabolic rate of ancient murrelets (IMR, *N* = 18 birds). The ambient temperature (*T*a) during our study was relatively constant because the boxes were buried in the ground and in deep shade, ranging between 4–9°C. Because ancient murrelets are very susceptible to nest desertion if disturbed [31], all activities that required handling birds outside the burrow occurred at the end of incubation. Consequently, only pairs which completed incubation were included in the analysis.

To measure fasting duration at the time of measurements (i.e. time since arrival at the nest box), one of each pair of study birds was equipped with a miniature radio transmitter (Pip Ag376∶1.3 g, LOTEK, St. John’s, NF, USA) and a metal band (if not already banded) without removing the bird from the nest box. Before measuring IMR, we left the radio-equipped birds undisturbed for at least three days.

### Incubation Metabolic Rate

**Figure 1:**
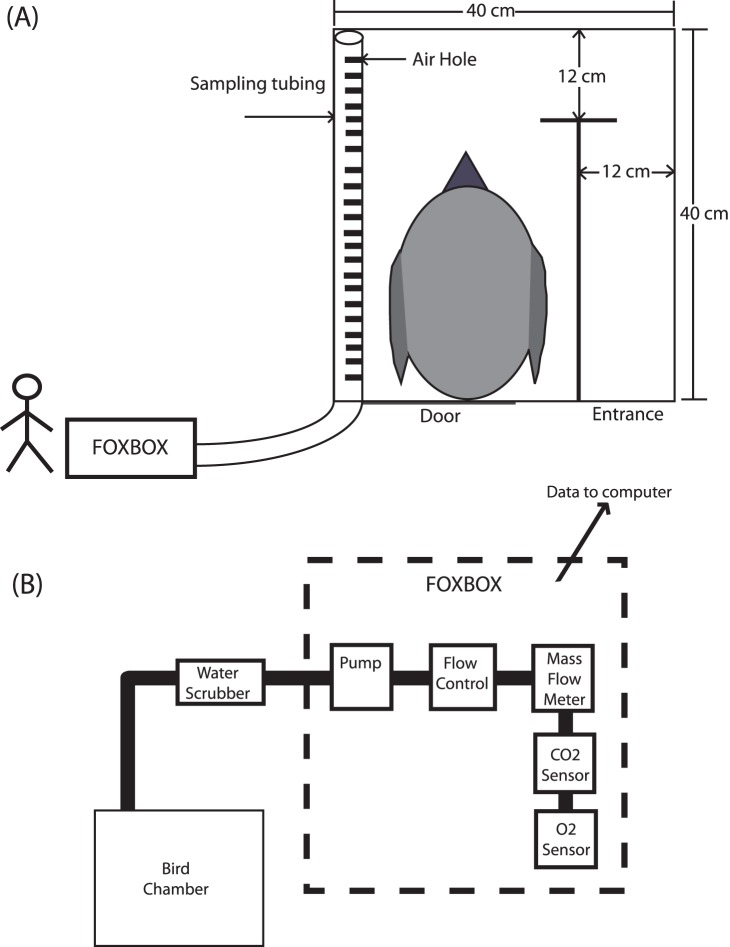
Schematic representation of a nest-box modified into a metabolic chamber (A) and airflow from a bird chamber to FOXBOX (B). Air pulled from a nest chamber into FOXBOX (A). Concentrations of CO_2_ and O_2_ inside a nest box were measured through FOXBOX (B).

We used open-flow respirometry to measure metabolic rates of naturally incubating birds in a respirometry chambers, which comprised modified artificial wooden nest boxes (external dimensions: 40 cm × 40 cm × 13 cm; [33]) buried in the ground. All the birds were sampled in artificial nest boxes. A sampling tube was inserted along one side of the main chamber of each nest box with air flowing through the chamber during the entire measurement (Fig. 1) and a FOXBOX II® (FoxBox, Sable Systems International, Las Vegas, NV, USA) pulled air from the sampling tube through the gas analyser’s sampling stream. All connections between the various components of the respirometry system were made with gas impermeable Bev-A-Line tubing and connectors. Prior to our recording, the equipment was tested with captive animals in the laboratory (i.e. Japanese quail *Coturnix japonica*). With this arrangement, the sampling tubes constituted part of the normal structure of the nest box, allowing us to take samples without disturbing the birds.

During each trial gas composition was recorded at 2–10 s intervals for 15 to 45 min after equilibrium had been achieved using Daemon software (Sable Systems International). We recorded the baseline gas levels (scrubbed of water, and, for the oxygen analyzer, of carbon dioxide) without a bird for 10 min before and after each trial, using a randomly selected empty nest box similarly placed in the ground. Prior to recording, we ran the machine until readings reached a plateau. Data were excluded from this analysis when no plateau was reached within 45 min.

Preliminary investigation showed that the washout constant was 5 min and flow rate was approximately double that needed to capture all outflowing air. We used magnesium perchlorate to dry outside air with a flow rate of ∼1.2 L/min, which was pumped through the chamber using the pump and flow meter built into the FoxBox II® respirometer. The effluent air passed through the FoxBox carbon dioxide analyser. Carbon dioxide was then removed from the air using soda lime and the effluent was passed through the FoxBox oxygen analyser. Both the oxygen and carbon dioxide analysers were calibrated at the start and end of each field season using pure nitrogen and 30% oxygen-70% nitrogen stock gas. The gas analysers were calibrated before each measurement using soda lime (CO_2_ scrubber). We corrected for analyser drift linearly during data analysis.

We measured both 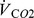 and 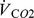. Because of substantial drift in the oxygen analyser associated with variation in ambient temperature and pressure in a field environment, we relied on 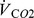 for comparisons across time. Nonetheless, the measurement of 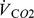 was highly correlated with the measurement of 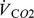 (*r* = 0.92, *n* = 58). We measured 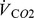 every four hours (04∶00, 08∶00, 12∶00, 16∶00, 20∶00 and 24∶00 Pacific Daylight Time: approximately 1.5 hours ahead of solar time at our field site) to examine diel variation in metabolic rates. We did not measure continuously for the 48 h period to allow birds to exchange incubation duties without disturbance. Change overs normally took place between 24∶00 and 04∶00. Total energy consumption of the embryo is only a small percentage (0.3–3%) of the energy expenditure of the parents during incubation [34], [35] and thus we assumed that egg metabolism made a negligible contribution to overall metabolism.

We used the automated drift correction function in ExpeData (Sable Systems) to account for analyser drift in our O_2_ and CO_2_ signals and then calculated 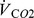 and 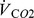 (mL min^–1^) for each individual using that software [36]. Studies that calculate energy expenditure based on CO_2_ production, such as doubly-labeled water methods, must assume a value for RQ (usually between 0.71–0.75 for resting birds: [37]), and reported energy expenditures are inversely related to those values. RQ can change significantly over the course of incubation, suggesting the possibility that changes in diet over the season can be misconstrued as changes in energy expenditure over the course of the season. In this study, we calculated RQ index as:
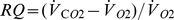



### Plasma CORT Analysis

To determine baseline CORT levels of adult ancient murrelets during breeding, we collected brachial blood samples from incubating murrelets at the end of incubation (May–June 2008). For each individual we preserved a drop of blood on filter paper to determine sex using PCR [5]. All blood samples were obtained within two minutes of capture. They were collected in heparinised cryovials and centrifuged immediately after collection. Centrifuged samples were kept frozen until prepared in the laboratory. CORT levels were measured using a specific and sensitive ^125^I double antibody radioimmunoassay (MP Biomedical kit 07-120103). The samples were measured in duplicate in a single assay (intra-assay variability = 5.4%; minimum detection level = 0.05 ng mL^−1^). Baseline corticosterone (i.e. samples collected within less than 3 min of capture [38]) should reflect the energetic state of a given individual, unaffected by the stress of capture [39].

### Statistical Analysis

All analyses were performed in R 2.15.2 [40]. We tested for normality in the distribution of all variables and log_e_-transformed those that were not normally distributed. For model fitting, we used response variables: residual of RQ on *T*a and CORT level respectively in two separate models. Fasting duration (short vs. long for RQ) and reproductive success (1 or 2 chicks for CORT) as explanatory variables. As all the pairs included laid two eggs and completed incubation, reproductive success was determined solely by hatching success. Data were collected repeatedly from each individual for IMR. To account for pseudoreplication, individual identity was included as a random effect in the model. For model fitting, we used generalized linear mixed models with the lmer function in the R lme4 package.

**Figure 2:**
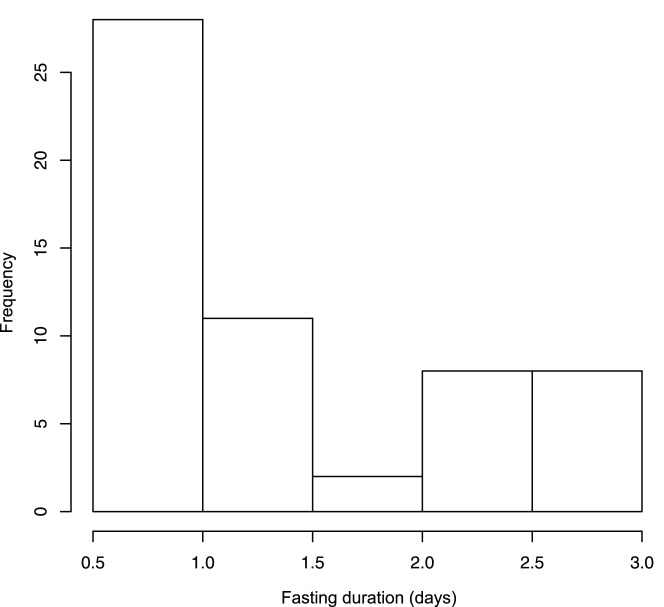
Frequency of fasting duration at the time of measurements of incubation metabolic rate in ancient murrlets at Reef Island in 2010.

To examine the relationship between the RQ index and fasting duration, measurements were divided into those made before (<2 d fasting at the time of measurements) and after (≥2 d fasting at the time of measurements) the modal fasting duration (Fig. 2) [5]; the same break point was also found using piecewise regression analysis. Clutch size of each pair was determined at the end of incubation and the status was confirmed after chicks departed to sea. Reproductive success was measured as number of chicks that successfully departed from the colony. Unless otherwise indicated, means ± SE are presented. CORT levels were compared between pairs with two chicks surviving to depart from the colony and pairs with only one chick departing.

## Results

**Figure 3:**
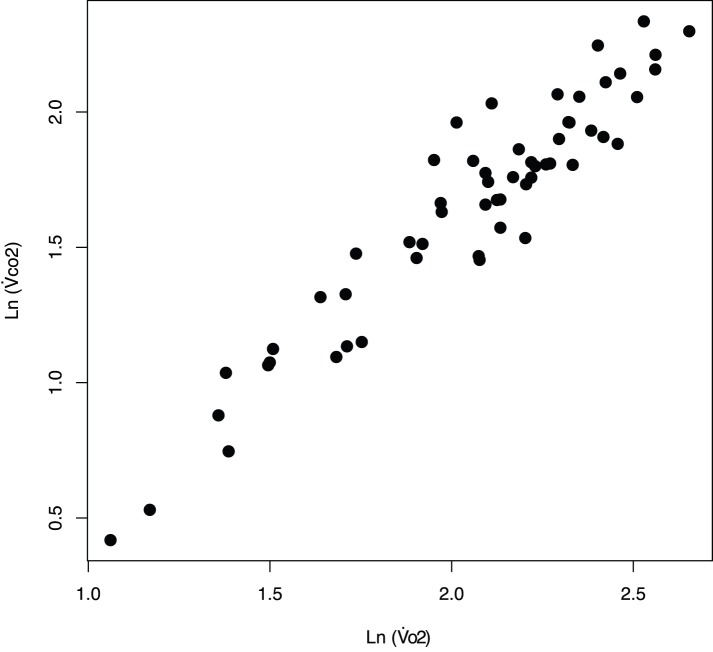
Relationship between Ln (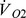: oxygen consumption rate) and Ln (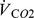: carbon dioxide production rate) in incubating ancient murrlets at Reef Island in 2010 (*N* = 18 birds, *N* = 58 measurements).

Ancient murrelets weighed 215±2 g at the end of the incubation period in 2010. Mean fasting duration at the date of weighing was 1.46±0.12 days (*N* = 18 birds, *N* = 58 measurements). Metabolic rates of incubating ancient murrelets were 1.57±0.07 mL CO_2_ g^−1^ h^−1^ ( = 17.32 J g^−1^ h^−1^, which translates to 25.8 kJ L 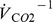, assuming a conversion coefficient of 25.8 kJ L 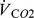; Fig. 3). There was no significant effect of body mass on incubation metabolic rates (*t*_57_ = 1.95, *P* = 0.06), although body mass variation was relatively low (coefficient of variation = 3.4%). 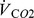 values were independent of mean fasting duration (*t*_57_ = 0.82, *P* = 0.42) and of reproductive success (*t_57_* = −1.07, *P* = 0.29). There was no difference in mean RQ between daytime and nighttime (*t_43_* = 0.04, *P* = 0.96). RQ increased with *T*a (R^2^ = 0.31, *t_57_* = 5.22, *P*<0.0001) and calendar date (R^2^ = 0.31, *t_57_* = 5.16, *P*<0.0001). RQ was higher for shift lengths ≥2 days than for shorter shifts (*t*_57_ = −11.21, P<0.0001; Table 1), and the difference was still significant after accounting for *T*a (residual of RQ on *T*a: *t*_57_ = −2.30, *P* = 0.02).

**Table 1:** The relationships between RQ and the length of fasting endurance, and CORT levels and reproductive success in ancient murrelets.

	Residual ofRQ on *T*a		CORT level
Short duration	−0.02±0.01	1 chick	23.25±6.60
	(*n* = 40 measurements)		(*N* = 6 birds)
Long duration	0.04±0.02	2 chick	12.69±1.04
	(*n* = 18 measurements)		(*N* = 32 birds)

**Figure 4:**
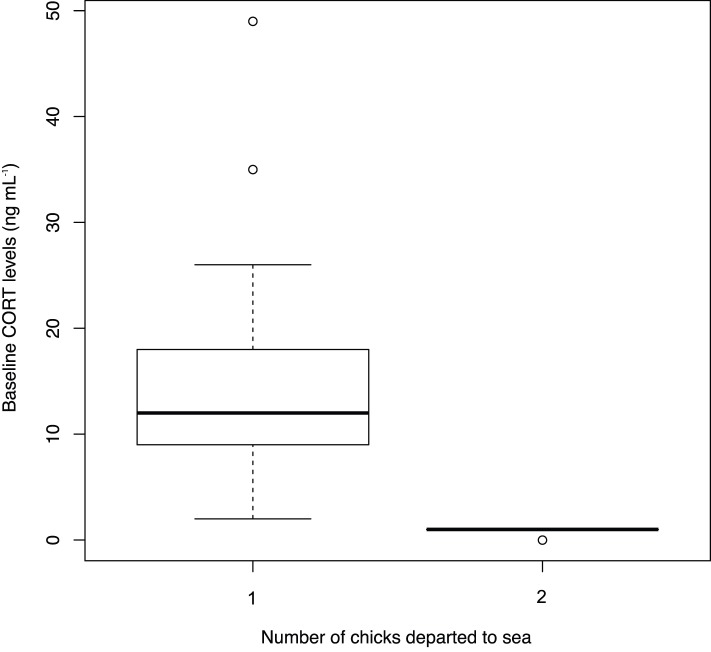
Baseline CORT levels (ng mL^−1^) of 38 ancient murrelets by number of chicks that departed to sea. All samples were taken at the end of incubation period at Reef Island in 2008.

In 2008, ancient murrelets weighed 200±2 g (*N* = 38 birds) and mean plasma CORT level was 13.8±1.4 ng mL^−1^ (range = 2 to 49 ng mL^−1^). There was no difference between males and females (males = 14.0±2.4 ng mL^−1^, females = 13.7±1.5 ng/mL, *t*_31_ = 0.11, *P* = 0.91). All pairs in this study incubated two eggs, but 6 out of 38 pairs had only one chick because one egg failed to hatch. The birds with two chicks had significantly lower levels of CORT (12.6±1.0 ng mL^1^, vs. 23.2±6.6 ng mL^1^, *t*_36_ = 5.40, *P*<0.0001; Table 1; Fig. 4). CORT levels were independent of mean fasting duration (*t*_18_ = −0.35, *P* = 0.72) and of hatching date (*t*_36_ = 0.11, *P* = 0.90).

## Discussion

We found birds that produced one chick exhibited higher CORT levels at the end of incubation than those birds that produced two chicks. The difference in CORT levels suggests that birds hatching only one chick had more difficulty in completing incubation than those that reared two chicks, presumably because they found it more difficult to secure the necessary nutrition. Thus, variation in CORT appeared to be associated with variation in reproductive success. As reproductive success was determined during incubation (via egg viability or hatching success) and CORT was measured at the end of incubation, a key assumption is that individual variation in CORT levels at the end of incubation is representative of individual variation in CORT levels earlier in incubation.

There is a strong body of literature showing that higher CORT levels usually coincide with lower foraging success in seabirds [41], [42], [43], [44], [45]. In our case, as CORT mobilizes glucose in the blood stream and is negatively correlated with food availability in seabirds at the colony [18], [19], [46] and individual [27] level, we suggest that the relationship between CORT level and reproductive success may indicate that birds hatching only one chick found it difficult to maintain adequate nutrition during incubation [4], [28], [30]. Furthermore, CORT may negatively interact with prolactin, a hormone that facilitates parental behavior [47], [48]. We propose that birds with low foraging success may have had higher levels of CORT and lower levels of prolactin, resulting in decreased reproductive success [49], [50]. Given the linkages between foraging success, incubation shift length and reproductive success in murrelets [5], we suggest that reduced foraging success in murrelets reduces investment in current reproductive success, partially mediated by CORT.

Our values for CORT were almost two orders of magnitude higher than a published value for the congeneric *Synthliboramphus hypoleucus* (0.2 ng mL^−1^, [51]). However, they were similar to other measurements in auks (both guillemot species *Uria* spp. *:* 1.5–7.6 ng mL^−1^, rhinoceros auklets *Cerorhinca monocerata*: 10 ng mL^−1^, least auklets *Aethia pusilla*: 6 ng mL^−1^ [52], [53]), which suggests that the measurement for *S. hypoleucus* is the outlier value.

RQ index provides an indication of the principal substrates being utilized [54]. In our study the initial RQ index indicated that, as expected, fat was the main energy resource during fasting for murrelets [55], [56]. RQ increased with date, perhaps indicating a change in energy sources from capital (less protein) to income (more protein) breeding [8]. Mass loss in ancient murrelets during incubation occurs linearly from clutch completion to hatching [31]. Also, incubation shift lengths of ancient murrelets tend to become shorter as incubation proceeds [5], [30]. These factors suggest that the long incubation shifts of ancient murrelets rely on initial body reserves, and when energy production shifts towards reliance on concurrent foods, murrelets need to reduce shift lengths. Thus, variation of shift length within this species may depend on initial body reserves.

After accounting for temperature, the RQ value was higher for birds that had been fasting longer. As discussed above, if fasting endurance is prolonged, metabolism may switch from primarily lipids, towards more protein [15], [16], [17], [57], as reflected in an increased RQ [58]. However, previous studies were based on penguins and albatrosses, which fast for much longer than murrelets during incubation. Also, murrelets are much smaller than penguins and albatrosses and therefore lipid reserves are more limited, although most body composition variation in auks is due to variation in lipids [59], [60], [61]. Possibly, RQ index increases during the longest fasts in murrelets because the birds shift into phase III fasting, when protein catabolism increases [15], [16], [17]. Alternatively, the phases may be less distinct in murrelets than in penguins and some catabolism may occur prior to the commencement of true phase III fasting.

To conclude, we suggest that incubating ancient murrelets shift from a lipid-only metabolism towards a partly protein-based metabolism as their incubation shifts exceeded two days. The increase in RQ with date implies a switch from reliance on capital (fat) to income (increasingly protein as the season progressed). High levels of plasma CORT were associated with birds that hatched only one nestling, indicating that elevated levels of CORT may have mediated reduced reproductive success. Due to the lack of correlation between CORT and incubation shift length, we suggest that those birds that undertook prolonged shifts did so only when they did not jeopardize their energy reserves.
